# The Effects of Interior Materials on the Restorativeness of Home Environments

**DOI:** 10.3390/ijerph20146364

**Published:** 2023-07-14

**Authors:** Jing Zhao, Yukari Nagai, Wei Gao, Tao Shen, Youming Fan

**Affiliations:** 1 School of Knowledge Science, Japan Advanced Institute of Science and Technology, Nomi 923-1292, Japan; 2 School of Art & Design, Dalian Polytechnic University, Dalian 116034, China; 3 College of Design and Innovation, Tongji University, Shanghai 200092, China; 4 School of Information Science, Japan Advanced Institute of Science and Technology, Nomi 923-1292, Japan

**Keywords:** restorative environment, interior material, Attention Restoration Theory (ART), restorative factor

## Abstract

The effects of a restorative environment on attention restoration and stress reduction have received much attention in societies, especially during the COVID-19 pandemic. Interior materials are a crucial environmental element influencing people’s perceived restorativeness at home. Nevertheless, few studies have examined the links between interior materials and the restorativeness of home environments. To address this gap, this study aimed to investigate the restorative potential of interior materials among a sample of adults in China. Cross-sectional data from 85 participants whose professional majors were related to interior design were selected. The measures of the restorative potential of each interior material were obtained by a questionnaire adapted from the semantic differential method. The Wilcoxon signed-rank test was used to compare the restorative potential of interior materials. We found that glass material had the best restorative potential in home environments. Doubts were raised regarding wood material’s restorativeness, and more consideration should be granted for designing a restorative home with wood material. In contrast, metal is not recommended for restorative home design. These findings contribute to the evidence of the restorative effects of home design.

## 1. Introduction

Numerous studies have highlighted several mental health problems, such as stress [1], depression [2], and anxiety [3], in modern society. Notably, the isolation caused by the COVID-19 pandemic was a trigger. People felt more stressed [4] and had more insomnia [5]. Therefore, there is growing scientific interest in how emotional reactions to environments, namely restorative environments, can alleviate mental problems [6]. The restorative environment can be defined as a substantive branch of environmental psychology related to recovery from mental fatigue [7]. Several studies have found that a restorative environment can benefit mental health [8,9,10]. For example, S. Kaplan et al. [11] found that people would feel calm, relaxed, and thoughtful after experiencing the restorative environment of a museum. In another study, Hartig et al. [12] compared different environmental categories and found that participants felt less anger and sadness in a restorative natural environment. Meanwhile, Cho et al. [13] found that the restorative environment in cultural heritage sites could improve cognitive functioning.

In a home environment, several individual elements, such as greenness [14], window view [15], furniture type [16], and interior material [17], were found to be related to restorativeness. Regarding the material, it has its own attributes that impact various aspects of occupants’ physical, psychological, and physiological status. Regarding physical health, the potential of a house’s dust pollutants from the surfaces of different materials is a health risk source for residents [18]. The interior surface material of polyvinyl chloride plastics was also found to be related to bronchial obstruction in young children in homes [19]. In addition to the physical health risks, wood material may convey positive psychological impacts (e.g., bright, pleasant, and warm) on occupants’ mental status [20] and has shown stress-reducing potential in interior design [21]. Unlike the positive effects of wood, white steel showed the negative impacts of material on psychological status. It might evoke impressions of being unhealthy and closed or even depressed [22]. The effects of different levels of wooden material quantity in rooms on physiology resulted in statistically disparate autonomic nervous system reactions [23]. Meanwhile, a particular wood category of Japanese cedar impacted occupants’ blood pressure levels [24]. Furthermore, materials are also connected with other performances, such as visual comfort quality [25], safety perception [26], and acoustic experience [27,28], the characteristics of which can also be considered as an effective source of restorativeness, such as noise isolation [29]. Together, these findings have shown the importance of interior materials in supporting the health and well-being of people.

Plenty of benefits of a restorative home environment have been acknowledged in the literature, such as a cabin home [30], a second rural home [31], and home attachment [32]. In addition, the frequency of experiencing a restorative environment could result in different health, working, and emotional satisfaction levels [33]. Since humans spend much of their time in their homes [34], the home environment may be ideal for restorativeness. As a result, applying restorative factors to the home, such as natural elements, is an effective design strategy for creating a restorative environment for our daily lives [35]. Although interior material is a critical factor in home restorativeness, this topic is limited to two important study topics. First, many previous studies have examined the positive restorative effects of wood materials in home environments. However, there is a lack of studies exploring the restorative effects of other materials in homes, such as textiles, glass, and brick. Second, although the benefits of using natural materials instead of artificial materials in home environments have been stated by a previous study [36] (pp. 78–80), the restorative effects of non-wood natural materials such as stone and soil are unknown. To address these gaps, this study aimed to investigate humans’ subjective perceptions and reactions to primary materials in interior design regarding restorativeness and discuss the potential of these interior materials for a restorative home environment. Based on this aim, several subsidiary research aims were addressed:

- To find the potential interior materials, which can show a positive effect on restorativeness, except wood material.
- To learn the restorative potential of other natural interior materials by comparing wood and other natural interior materials.
- To systematically compare commonly used interior materials for restorative home environments.

## 2. Methods

### 2.1. Study Design

**Figure 1:**
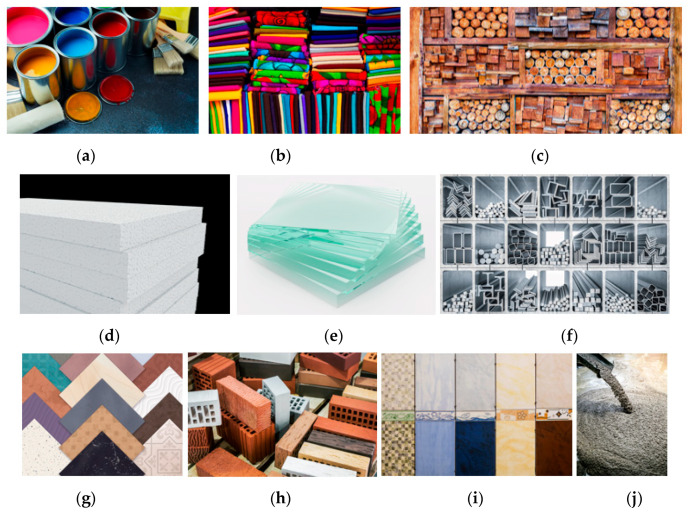
Pictures of interior materials for investigation: (**a**) interior wall paint; (**b**) textile; (**c**) wood; (**d**) plastic; (**e**) glass; (**f**) metal; (**g**) tile; (**h**) brick; (**i**) stone; (**j**) concrete. Source: free and premium license from Freepik.

According to a professional and required textbook which comprehensively introduces materials and specifications used in interior design, many material categories are used in the interior design field [37]. In addition, we used the content analysis of the literature, removing materials rarely used in the interior home environment. We categorized interior materials into ten different categories as the investigating target in this study. They were interior wall paint, textile, wood, plastic, glass, metals, tile, brick, stone, and concrete. The images of each material are shown in Figure 1.

We used the semantic differential (SD) method to investigate humans’ subjective perceptions and reactions to these ten materials. The reliability of the SD method in measuring peoples’ subjective attitudes to interior material has been reported elsewhere [38,39,40]. A material evaluation attribution system developed by Bhise et al. [41] was applied to the adjective pairs in the SD method to measure the perception of interior material quality for automotive interior design. It contains physical, attribute, and evaluative classes, each with multiple variables to express its properties.

According to restoration theory, four features of a restorative environment, including ‘being away’, ‘extent’, ‘fascination’, and ‘compatibility’, were chosen based on previous study [42]. In addition, several previous studies have reported the reliability of these items in measuring restorative potential [43,44]. Combining the expounding on these four features from Herzog et al. [45] with the material attribution, we selected adjective words for four features’ evaluation from the material evaluation attribution system. Being away means the environmental contents could elicit different mental conditions from what is ordinary. This feature refers to the atmosphere that is posed by the environment. Therefore, we selected the adjectives that best related to the atmosphere feeling from the evaluative variables (ordinary–special, harmony–clash). Extent means that the environmental contents are sufficient to occupy the mind. From the point of the material, this feature can be found from the appearance. We selected some adjectives from the appearance variables (textured–untextured, colorful–dull, finished–unfinished, patterned–random). Fascination is the environmental ability to hold an occupant’s attention without effort, and three adjective pairs were selected for this feature (luxury–cheap, pleasing–repelling, attractive–unattractive). Compatibility is the range of support the materials provide. We used three adjective pairs (durable–nondurable, solid–flimsy, changeable–unchangeable) from the physical variables to express this feature. All of the features and adjective pairs are shown in Table 1.

### 2.2. Measures and Participants

The questionnaire was posted through an Internet platform (https://www.wjx.cn/) and consisted of 12 adjective pairs with 1–7 scales to rank the level of attitude toward the materials. Here, 1 represents the weakest feeling about this item; 7 represents the strongest feeling about this item. Material is an abstract term for evaluating the component of environmental restorativeness. Same material can be used for various areas and purposes, which may express different properties. To show the material properties and obtain accurate impressions when they appear in home environment, we selected the participants with interior-design-related major or career. First, interior-design-relevant participants are experts who are familiar with the properties of each material when using them in home environment. Second, they are experienced in the treatments and expressions of each material from the material to a home environmental design. While answering the questionnaire, participants were asked to view the pictures and utilize information about the materials from the Internet or their own experience to fill in the 12 adjective items they perceived about the material’s properties. There were 10 materials, each with 12 adjective items to evaluate. Participants took approximately 30 min to complete the questionnaire.

Before answering the questionnaire, participants read and filled in the consent form. It described the content of the investigation and that there was no risk in answering this questionnaire. Furthermore, participants were informed that all the data would be used strictly for this research, and they were able to log out or quit at any stage. We collected a total of 85 answer sheets, and the participants’ majors/careers were environmental design students, interior design students, and architects in Chinese university or design company.

### 2.3. Statistical Analysis

Before analyzing the difference between the interior materials, the normality of these data between every two materials was tested. The *p*-values of the Kolmogorov–Smirnov tests were less than 0.05, meaning that the paired sample T test could not be used for analyzing variances. Therefore, the Wilcoxon signed-rank test was selected to analyze the matched-pair variances [46]. Each restorative feature index (being away, extent, fascination, compatibility) was calculated using its adjective items (means value of their adjective items). To compare each 2 interior materials out of the 10 categories (interior wall paint, textile, wood, plastic, glass, metal, tile, brick, stone, and concrete), a total of 45 pairs were compared. All statistical tests were conducted in SPSS (https://www.ibm.com/products/spss-statistics (accessed on 25 February 2023)), and the significance level was set at *p* ˂ 0.05 (2-tailed).

According to the questionnaire setting of being away, the high score represents the properties of ordinary and harmony. However, the definition of being away is how different you feel between the material’s design image and your ordinary environment. That means the score is lower, and the higher level of the being away feature of this material is shown. For the other 3 features (extent, fascination, and compatibility), the high score means the high extent, fascination, and compatibility levels. In addition to restorative potential of these 4 features, Laumann et al. [44] found the features of compatibility and fascination could predict a self-rating aspect of preference, and being away and compatibility could predict aspect of relaxation. Therefore, we added these two aspects (preference and relaxation) as supplementary indicators of restorative potential to show the difference. In total, we compared three aspects of each pair of materials:

- Preference: calculated by the total value of fascination and compatibility.
- Relaxation: calculated by the total value of being away and compatibility.
- Restorative potential: calculated by the total value of being away, extent, fascination, and compatibility.

After summarizing the Wilcoxon signed-rank test outputs, the material pairs with statistically significant differences in preference, relaxation, and restorative potential were obtained. Then, by combining them with the data of means and medians, the rank relationships of these ten material categories became transparent.

## 3. Results

After obtaining scores of being away, extent, fascination, and compatibility, the mean and median values were calculated to determine materials’ preliminary ranks for each restorative potential feature (Table 2). In the feature of being away, wood and glass showed excellent scores, while metal was ranked last. In the feature of extent, the rank from best to worst was concrete, metal, stone, brick, plastic, glass, interior wall paint, wood, tile, and textile. For fascination, the highest score was plastic, and the lowest was wood. The compatibility feature ranked glass, plastic, textile, tile, brick, concrete, stone, interior wall paint, metal, and wood. From the results of the Wilcoxon signed-rank test, the details of two materials with statistically significant differences could be obtained based on *p*-values. We calculated four features of 45 material pairs and summarized the results in Table 3. Table cells with bold font of *Z*-value and an asterisk (*) have statistically significant differences.

Combining the mean values of each material and the results of the Wilcoxon signed-rank test, we found statistically significant differences in restorative potential features of each material pair. Then, we summarized their results, and the ranking relationships of relaxation, preference, and restorative potential were obtained (Table 4). From the viewpoint of relaxation, glass had a better level than tile, brick, stone, concrete, and metal, and metal had a lower level than tile. From the point of view of preference, the score of plastic was better than metal, tile, interior wall paint, stone, and wood. The score of brick was better than interior wall paint, metal, and stone. The level of wood was lower than glass, tile, brick, concrete, and textile. From the viewpoint of restorative potential, the score of concrete was better than metal.

## 4. Discussion

This was one of the few studies that examined the effect of interior material on restorative potential using a sample of interior-design-relevant participants in China. We found that the glass material ranked the best, statistically better than the tile, brick, stone, concrete, and metal materials in the aspect of relaxation. The being away feature of glass was excellent, and it had the best score in compatibility. The mean value of relaxation was higher than all other materials in this investigation. From the aspect of preference, although glass was only statistically better than wood, it was not statistically worse than any other materials in the aspects of preference and restorative potential. This finding indicated that glass material showed a positive result of restorative potential. Actually, the material of glass is related to many environmental components in our daily life [47], and some evidence has shown that glass is connected to restorative effects. For example, the window view of nature could convey positive effects on apartment residents’ satisfaction and well-being [15]; a window view to the sky has been found to benefit attention restoration for densely populated cities’ residents [48]. Additionally, the glass window is one way of enjoying daylight, which is the best light source and is connected with occupants’ psychophysiological well-being [49]. Furthermore, some studies have shown a high aesthetic impact of glass material [50]. They have discussed glass as an attractive building material [51] and structure [52] that has been used daily for decades. A facial micro-reaction analysis found that participants felt positive when facing glass structures of walls, facades, and roofs [53]. Although glass structures are sometimes connected with an image of building vertigo because of their properties and the rise of high-rise buildings [54], plenty of places actively produce a desire to experience the building of vertigo [52]. Moreover, several criteria have also been discussed for comfort-driven glass design and have shown the potential of a glass environment [55].

The secondary finding of this study was that wood might not be the best material for restorative potential in a home environment. From the aspect of preference, wood scored less than plastic, stone, glass, tile, brick, concrete, and textile. Although wood received the best score in the feature of being away, the scores of extent, fascination, and compatibility were not good. This was in contrast with some previous studies. For example, Watchman et al. [20] found that a wood room often conveys the impression of bright, pleasant, and warm compared with a no wood room. Sakuragawa et al. [22] found that wooden walls could connect people to nature. Nevertheless, some negative impacts still exist in wood-related topics, such as emitting volatile organic compounds, formaldehyde, and acrolein, which pose health risks [56] as well as weathering [57] and the adverse effects of a hygroscopic material [58]. Therefore, Strobel et al. stated that the difficulty of maintaining interior wood material was the most frequently mentioned reason for not using wood [59]. Furthermore, a study confirmed that different gender, background, and culture could lead to disparate wood design and preferences; hence, wood material use may be driven by multi-criteria [60]. In summary, a review of interior wood material mentioned that the details of wood types and attributes expected to benefit from restorative effects are still in question [21]. More restorative environmental tests with strong wooden material designs and specific criteria are needed to discuss this gap.

From the relaxation aspect, metal had the worst mean score and was statistically less than glass and tile. Moreover, its score was less than plastic and brick in preference. Furthermore, metal was worse than concrete in all features of restorative potential, it received the worst score in being away and a low score in compatibility. This result was consistent with the image of metal from previous studies. For instance, Sakuragawa et al. found that white steel may convey images of being unhealthy, closed, and even depressed [22]. According to a human cerebral blood flow study, the stimuli from metal evoked a stress response and gave participants the impression of cold, hard, and artificial [61]. Even amongst metal material packs, it could convey an image of a lower price to change the consumer’s perception of willingness to buy [62]. These may be why a finding from building materials’ visual and tactile evaluation showed that steel’s sensory descriptions and expressive meanings are cold, industrial, and unpleasant [63]. Therefore, the metal was described as ‘while simplicity, solitude, and stress-induced demonstrated the tangle of metal that can affect the application and achievement of desired living space’ from a semantic investigation of metal [39].

This study had several limitations in terms of its process and results. First, the target of this study was materials that were not the specific images of the design. It showed the difficulty in catching an accurate impression from the process material to the design. Although the participants were interior-design-related, some were students whose design experience could have been richer. Second, different regions have different design expressions, such as European design style, Southeast Asian design style, and Japanese style. Chinese participants completed this whole investigation. The design images of the material may be limited to the style in China. In addition, we asked the participants to search the information about the materials online. The evaluation may lead to bias depending on the search results. These actions open the possibility for perception gaps. Third, we planned to find people’s perceptions and reactions to interior materials. If the number of participants was higher, the results might be transparent and robust in expressing restorative potential. Finally, regarding the results, the detailed restorative potential of interior wall paint, textile, plastic, tile, brick, stone, and concrete could not be found. Future studies may be narrowed down to one or two material comparisons, for example, exploring more details about wooden interior design with more specific wood categories or attributes for restorative homes. Furthermore, according to the results of some material semantic roles, brick conveyed nostalgia, traditionality, and warmth [39], and some textile materials showed smoothness, softness, and elegance [64]. Future studies should focus on emotional reactions to brick or textiles and compare them with other materials, which may determine their potential for a restorative home environment. Although some shortages existed, the strength of this study was in attempting to explore the restorativeness effects of commonly used interior materials by the professional interior-design-related samples. It compared several interior materials regarding restorative potential and highlighted the interior material that could be enhanced in residential interior design. The results showed the potential of materials for restorativeness and may illuminate good guidelines for designing a restorative home environment. We also call for more concerns about emotional reactions to materials.

## 5. Conclusions

This study showed that glass material positively affects the restorativeness of a home environment. Restorative environmental designers could pay more attention to design expression based on glass. Although some research indicated the positive effects of wood material for restorativeness, its specific categories and designs need more discussion and consideration. Moreover, metal material may not be recommended for exposure in a restorative home environment design. Further studies in other environments and contexts are needed to confirm these findings.

We determined the restorative potential of materials primarily used in interior design. The interior designer, architect, or environmental designer could apply these findings to consider materials used to create a restorative home environment. Moreover, this study indicated some gaps in the literature between the emotional reaction to interior materials and restorativeness. In addition, the research concern of emotional reactions to materials was stated, which may inspire future researchers interested in this topic.

## Figures and Tables

**Table 1:** Restorative environmental features and adjective pairs.

Being away	Extent	Fascination	Compatibility
Ordinary–Special	Textured–Untextured	Luxury–Cheap	Durable–Nondurable
Harmony–Clash	Colorful–Dull	Pleasing–Repelling	Solid–Flimsy
	Finished–Unfinished	Attractive–Unattractive	Changeable–Unchangeable
	Patterned–Random		

**Table 2:** Means and medians of restorative features obtained from the ratings of 10 interior materials.

	Interior Wall Paint	Textile	Wood	Plastic	Glass
	M(Median)	M(Median)	M(Median)	M(Median)	M(Median)
Being away	3.25(3.50)	3.48(3.50)	2.95(3.00)	3.51(4.00)	3.07(3.50)
Extent	3.83(4.00)	3.45(3.75)	3.66(3.75)	3.92(4.00)	3.84(4.00)
Fascination	3.35(3.67)	3.29(3.33)	3.10(3.33)	4.24(4.33)	3.47(3.67)
Compatibility	3.05(3.00)	3.70(4.00)	2.94(3.00)	3.75(4.00)	3.90(3.67)
	**Metal**	**Tile**	**Brick**	**Stone**	**Concrete**
Being away	3.64(3.50)	3.39(3.50)	3.58(3.50)	3.53(3.50)	3.42(3.50)
Extent	4.18(4.00)	3.54(3.75)	3.92(4.00)	4.04(4.00)	4.48(4.25)
Fascination	3.79(4.00)	3.65(4.00)	4.20(4.33)	3.73(4.00)	4.18(4.33)
Compatibility	3.03(3.00)	3.39(3.67)	3.39(3.33)	3.18(3.33)	3.27(3.33)

**Table 3:** The Wilcoxon signed-rank test results of material pairs.

		Textiles	Wood	Plastic	Glass	Metal	Tile	Brick	Stone	Concrete
**Interior wall paint**	Being away	−1.32	**−2.29** *	−1.60	−1.35	−2.55 *	−1.18	−2.23 *	−1.69	−1.10
	Extent	−2.75 *	−1.48	−0.61	−0.04	−2.78 *	−2.74 *	−0.61	−0.88	−4.69 *
	Fascination	−0.40	−1.72	−5.83 *	−1.40	−3.30 *	−3.08 *	−5.78 *	−2.97 *	−5.14 *
	Compatibility	**−4.32** *	−0.77	−4.36 *	−5.03 *	−0.09	−2.48 *	−2.27 *	−1.14	−1.44
**Textiles**	Being away		−3.59 *	−0.25	−2.40 *	−0.96	−0.59	−0.78	−0.23	−0.40
	Extent		−1.48	−3.11 *	−2.95 *	−4.35 *	−0.96	−3.57 *	−3.87 *	−5.41 *
	Fascination		−2.02 *	−6.19 *	−1.73	−3.65 *	−3.28 *	−5.72 *	−3.53 *	−5.50 *
	Compatibility		−5.64 *	−0.38	−1.32	−4.34 *	−2.66 *	−2.92 *	−3.46 *	−2.74 *
**Wood**	Being away			−4.38 *	−1.21	−4.77 *	−4.16 *	−4.80 *	−3.83 *	−3.22 *
	Extent			−2.29 *	−1.78	−3.76 *	−1.21	−2.72 *	−3.03 *	−5.64 *
	Fascination			−6.88 *	−3.81 *	−5.23 *	−4.70 *	−6.55 *	−5.40 *	−6.25 *
	Compatibility			−5.49 *	−6.18 *	**−0.81**	−3.72 *	−3.38 *	−2.29 *	−2.18 *
**Plastic**	Being away				−2.75 *	−0.83	−1.26	−0.69	0.00	−0.86
	Extent				−0.81	−2.32 *	−3.42 *	−0.03	−1.09	−4.42 *
	Fascination				−5.36 *	−4.02 *	−4.62 *	−0.45	−3.97 *	−1.03
	Compatibility				**−0.78**	−4.68 *	−2.66 *	−2.63 *	−4.08 *	−3.16 *
**Glass**	Being away					−4.13 *	−2.59 *	−3.83 *	−2.47 *	−2.42 *
	Extent					−2.81 *	−3.06 *	−0.82	−1.99 *	−5.33 *
	Fascination					−2.54 *	−1.63	−5.31 *	−2.55 *	−5.81 *
	Compatibility					−5.48 *	−4.47 *	−3.54 *	−5.04 *	−4.11 *
**Metal**	Being away						−2.14 *	−0.36	−1.01	−2.10 *
	Extent						−4.46 *	−1.77	−0.95	−2.45 *
	Fascination						−1.23	−3.63 *	−0.24	−3.95 *
	Compatibility						−3.28 *	−3.37 *	−1.58	−2.36 *
**Tile**	Being away							−2.18 *	−0.86	−0.01
	Extent							−3.02 *	−3.81 *	−5.98 *
	Fascination							−4.45 *	−0.55	−4.11 *
	Compatibility							−0.23	−2.05 *	−1.27
**Brick**	Being away								−0.67	−1.84
	Extent								−0.95	−4.11 *
	Fascination								−3.85 *	−0.56
	Compatibility								−2.03 *	−0.89
**Stone**	Being away									−0.54
	Extent									−3.76 *
	Fascination									−4.02 *
	Compatibility									−0.85

* *p* < 0.05.

**Table 4:** Ranking relationships of interior materials which have statistical difference.

**Relaxation** (being away, compatibility)	Glass ˃ Tile ˃ Metal Glass ˃ Brick Glass ˃ Stone Glass ˃ Concrete	
**Preference** (fascination, compatibility)	Plastic ˃ Metal Plastic ˃ Tile ˃ Interior wall paint Plastic ˃ Stone ˃ Wood	Glass ˃ Wood Tile ˃ Wood Brick ˃ Wood Concrete ˃ Wood Textile ˃ Wood
	Brick ˃ Interior wall paint Brick ˃ Metal Brick ˃ Stone	
**Restorative potential**	Concrete ˃ Metal	

## Data Availability

The data generated and/or analyzed during the current study are not publicly available but are available from the corresponding author on reasonable request.
